# Transcriptomic comparison of primary bovine horn core carcinoma culture and parental tissue at early stage

**DOI:** 10.14202/vetworld.2017.38-55

**Published:** 2017-01-13

**Authors:** Sharadindu Shil, R. S. Joshi, C. G. Joshi, A. K. Patel, Ravi K. Shah, Namrata Patel, Subhash J. Jakhesara, Sumana Kundu, Bhaskar Reddy, P. G. Koringa, D. N. Rank

**Affiliations:** 1 Veterinary Officer (WBAH & VS), West Bengal Animal Resources Development Department, Bankura - 772 152, West Bengal, India; 2 Department of Animal Genetics & Breeding, College of Veterinary Sciences and Animal Husbandry, Anand Agricultural University, Anand, Gujarat, India; 3 Department of Animal Biotechnology, College of Veterinary Sciences and Animal Husbandry, Anand Agricultural University, Anand, Gujarat, India; 4 Hester Biosciences Limited, Ahmedabad, Gujarat, India; 5 Veterinary Officer, MVC Sarenga, Government of West Bengal, Bankura, West Bengal, India

**Keywords:** cummerbund, gene ontology, primary culture, RNA-sequencing, squamous cell carcinoma of horn, transcriptome profiling

## Abstract

**Aim:**

Squamous cell carcinoma or SCC of horn in bovines (bovine horn core carcinoma) frequently observed in *Bos indicus* affecting almost 1% of cattle population. Freshly isolated primary epithelial cells may be closely related to the malignant epithelial cells of the tumor. Comparison of gene expression in between horn’s SCC tissue and its early passage primary culture using next generation sequencing was the aim of this study.

**Materials and Methods:**

Whole transcriptome sequencing of horn’s SCC tissue and its early passage cells using Ion Torrent PGM were done. Comparative expression and analysis of different genes and pathways related to cancer and biological processes associated with malignancy, proliferating capacity, differentiation, apoptosis, senescence, adhesion, cohesion, migration, invasion, angiogenesis, and metabolic pathways were identified.

**Results:**

Up-regulated genes in SCC of horn’s early passage cells were involved in transporter activity, catalytic activity, nucleic acid binding transcription factor activity, biogenesis, cellular processes, biological regulation and localization and the down-regulated genes mainly were involved in focal adhesion, extracellular matrix receptor interaction and spliceosome activity.

**Conclusion:**

The experiment revealed similar transcriptomic nature of horn’s SCC tissue and its early passage cells.

## Introduction

Cancer cell lines, in general, are used as a model in testing of anticancer drugs presently used [1,2] as well as in the development of new therapies [3,4]. There is no bovine cell line of squamous cell carcinoma (SCC) origin. This is probably the first ever attempt to develop a SCC cell line of bovine origin. The horn cancer-based cell line can be used as an *in vitro* model in cancer research to define potential molecular markers as well as for the screening and characterization of cancer therapeutics similar to human lung and breast cancer cell lines [5,6]. The results of the research in cancer cell lines can usually be extrapolated to *in vivo* tumors originated from squamous cells. Transcriptomic profiling of the initial passage cells and the SCC tissue was attempted in this study to confirm the initial passage cells represent the SCC tissue at molecular level.

Historically, *in vitro* cultures of SCC of horn (bovine horn core carcinoma [BHCC]) have been limited in availability and scope, compared to those from many other organs such as mammary tumors and endometrial cancer cell lines. Cell lines, those derived from metastases, do not span the range of most of cancer phenotypes, and in particular, are not representative of original SCC [7]. Furthermore, how extensively long-term culture alters the biological properties of cell lines are always of concern [8]. Adaptation of fresh cancerous tissue specimens which grow *in vitro* as primary cell cultures provides homogeneous cellular material, enriched in tumor cell component [7] and it also retains phenotypic, transcriptomics profile of the corresponding tissues from which they derive [8-10] at the first passages.

Usually, up regulations of genes are involved in proliferation and metabolism. Cellular activity within a tissue is evinced by the transcriptome at a specific time. Pathophysiology of complex diseases, like cancer, can be evaluated by an unbiased method like genome-wide expression studies [10]. RNA sequencing (RNA-Seq) analysis is an affordable accurate and comprehensive tool to analyze transcriptome of complementary DNAs (cDNA) using next generation sequencing (NGS), followed by mapping of reads onto the reference genome making it possible to identify introns, exons, their flanking regions and thus providing an opportunity to understand the complexity of eukaryotic transcriptome [11].

SCC of horn of bovines is a SCC of horn core mucosa with least known genetic landscape, reported only in *Bos indicus*. This causes heavy economic losses due to subsequent metastasis and death of animal. In India, approximately 1% of the cattle population is affected by this tumor [12], most commonly in working bullocks, sometimes in cows and rarely in bulls, buffaloes, sheep, and goats [13-16]. The incidence of SCC of horns is more frequent in Kankrej breed than other zebu cattle, crossbred or non-descript cattle [17]. From Sumatra [18], Brazil [19], and Iraq [20] few cases were reported. Till date, the comparison of gene expression profile between cell culture and parental tissue of SCC of horn of bovines has not been performed. The study was designed to compare gene expression profiles in SCC affected horn tissue and primary cell culture derived from that tumor using Ion Torrent PGM sequencing platform.

## Materials and Methods

### Ethical approval

Aprroval for research work granted vide approval no. IAEC: 155/2011 of College of Veterinary Science and animal Husbandry, Anand Agricultural Universuty, Anand-388 001, Gujarat.

### Tissue collection

Carcinomatous and normal horn core mucosa were collected during corrective surgery in RNAlater^®^ (Thermo Fisher scientific, Massachusetts, USA) from clinically affected (left horn) and normal (right horn) horn of a Kankrej breed of bullock (age 7 years) from Rajkot, Gujarat, India. Necrotic tissues were not collected. Fresh tissues were cut into pea-sized segments and preserved in:

- 10% neutral buffered formalin for histopathological studies
- RNAlater^®^ (Sigma-Aldrich, St. Louis, USA) for RNA extraction
- Dulbecco’s modification of Eagle’s medium (DMEM) (50 ml) (Thermo Fisher Scientific, Massachusetts, USA) with penicillin-streptomycin (500 µl) (Thermo Fisher Scientific, Massachusetts, USA) + amphotericin-B (500 µl) (Thermo Fisher Scientific, Massachusetts, USA) and brought to lab at 0-4°C.

### Histopathology

Horn SCC tissues were processed for histopathological studies and paraffin-embedded sections were cut at 5-6 µ thickness with section cutting machine (Leica, Germany) and stained with hematoxylin and eosin (H and E) [21]. The H and E stained sections were observed under light microscope and lesions were observed [21].

### Cell culture

After removal of adipose tissue, tumor tissues (at 4°C) were mechanically minced in 1 mm^3^ fragments. Then, the primary culture was established and incubated at 37°C and 5% CO_2_ [21]. Similarly, tumor tissue explant culture was also performed by standard protocol [16]. DMEM and Ham’s F12 50/50 mix (DMEM-F12) medium was changed twice weekly and split ratio for cells were 1:3 when cells reached up to 90% confluence. Cell morphology was observed in contrast phase, at 40× magnification, by inverted microscope. The cells were sampled at intervals, resuspended in a freezing medium (80% DMEM, 10% fetal bovine serum, and 10% dimethyl sulfoxide), and stored at −80°C at every two passages for cryopreservation.

Differential trypsinization was used for removal of the fibroblasts which detached sooner than the tumor cells. Isolation of pure population of tumor cells was done by plating approximately 10,000 detached cells in 100 mm Petri dishes and following dilution cloning [22]. These isolated clones were used for RNA-Seq purposes.

### Cell proliferation and doubling time assay

Two counts were performed for each passage, in triplicate. For doubling time analysis, plating of cells in triplicate onto 6-well plates at a concentration of 2.5 × 10^4^ cells/well in DMEM-F12 were done. After 24, 48 and 72 h, cells were collected after trypsinization and counted in a Neubauer chamber. Doubling time (in hour) was calculated as described in a previous study [23].

### RNA isolation

TRIzol (Sigma-Aldrich, St. Louis, USA) method as per manufacturer’s instructions was used to isolate RNA from early passage cells of SCC of horns (pooled RNA of passage 2 and 3) and parental SCC tissue.

### Preparation of sample and transcriptome procedure

All the protocols starting from mRNA isolation to library preparation were followed as per manufacturer’s instructions. The detailed protocol steps can be accessed from Ion Torrent’s “Ion Total RNA-Seq Kit” (Part No.: 4467098) using 316 chip.

### *In silico* gene expression analysis

Sequence reads were generated from cDNA libraries of early passage cells and parental SCC horn tissue using Ion Torrent PGM chemistry using 316 chips [24]. Raw sequence reads (*.fastq files) were checked for quality control in FastQC v0.10.1. To avoid low quality data negatively influencing downstream analysis, the reads were trimmed and low quality sequences were filtered using PRINSEQ-lite version 0.20.2 with default parameters in Linux. This quality checked reads were aligned to the bosTau7.fa build of the cow genome (http://hgdownloadtest.cse.ucsc.edu/goldenPath/bosTau7/chromosomes/) using GMAP [25] and Samtools allowing for unique non-gapped alignments to the genome. The default parameters for the GMAP method were used.

The resultant *.sam files were converted to *.bam files with Samtools then *.sorted.bam files were used in Cufflinks v 2.2.1. The resulting Cufflinks assemblies of all samples were combined together using Cuffcompare v 2.2.1. The differential expression was calculated by Cuffdiff based on transcript abundances [26]. Cuffdiff v 2.2.1 was then employed on the combined transcripts to identify differentially expressed genes/transcripts.

### RNA-Seq data normalization

The raw RNA-Seq read counts for cufflinks transcripts were first log_2_ transformed at fragments per kilobase of exon per million reads mapped (FPKM) and then quantile normalized.

### Functional annotation

The genes differentially expressed in SCC horn tissue and the short-term primary culture was selected for functional categorization. The comparisons between expressed genes which produced Cuffdiff output with “Q value” <0.01 and “OK” marked test status were considered to be differentially expressed. Gene ontology (GO) and pathway analyses of up and down-regulated genes by DAVID database [27] and PANTHER database [28] were done, respectively. Gene set analyses were done in terms of biological processes, molecular function, and cellular component. The list of differentially expressed genes having >5 FPKM value and log_2_ fold change value above 2 (based on FPKM ratio), p=0.05 and false discovery rate (FDR) value 5% were chosen.

Whole transcriptome analysis using NGS will identify several thousands of genes which are deregulated in number of cancer-related pathways. Since the depth of sequencing for each gene varies because of inherent methodology involved in NGS, it is globally accepted protocol to validate data obtained by this methodology via randomly selecting few of the genes through quantitative real-time polymerase chain reaction (PCR) [29,30]. Since it is practically impossible to validate all of the genes found in NGS-based study as well as it is economically non-feasible approach to study all identified genes, we have followed standard procedure to validate NGS data by selecting randomly selected sufficiently large set of transcripts and proved concordance of expression pattern using quantitative real-time PCR (Data not shown).

## Results

### Histopathology of SCC tissue

**Figure-1:**
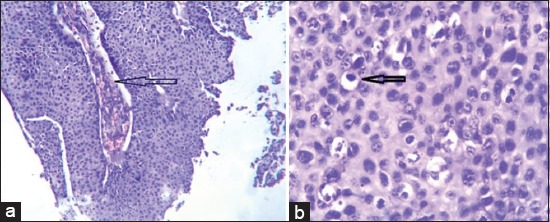
(a) Keratinization of individual horn squamous cell carcinoma (SCC) epithelial cells of parental tissue as seen in H and E stain at 100×, (b) pleomorphic horn SCC cells with nucleolar polymorphism of parental tissue as seen in H and E stain at 100×.

The tumor cells were tightly cohesive, featured with moderately high to abundant eosinophilic cytoplasm. The nucleus to cytoplasmic ratio was potentially increased with nuclei showing frequent prominent nucleoli. Mitotic activity was abundant including atypical forms such as ring and tripolar configurations. Intercellular bridges were focally present. Keratinization of individual epithelial cells (Figure-1a) and pleomorphic epithelial cells with enlarged nuclei (Figure-1b) were seen. Histopathology confirmed SCC of the horn core epithelium.

### Isolation of SCC horn epithelial cells

**Figure-2:**
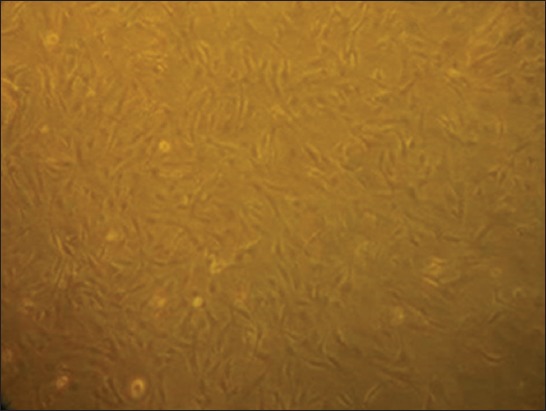
Primary monolayer culture of horn squamous cell carcinoma cells at 40×.

**Figure-3:**
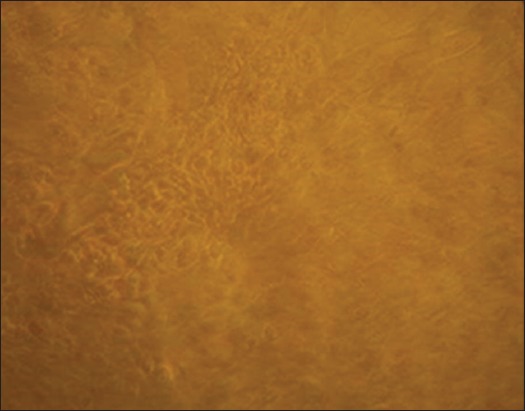
Rounded up horn squamous cell carcinoma malignant early passage cells on day 7 at 40×.

Primary monolayer culture with finite mitotic lifespan (SCC early passage cells) was established from the bullock affected with SCC of horn (Figure-2) following the enzymatic disaggregation methods as described earlier [22]. By the first week, tumor cells were seen rounding up and growing throughout the T-25/T-75 flask (Figure-3) among the normal stromal fibroblasts that grew in parallel.

### Growth curve and population doubling time analysis

**Figure-4:**
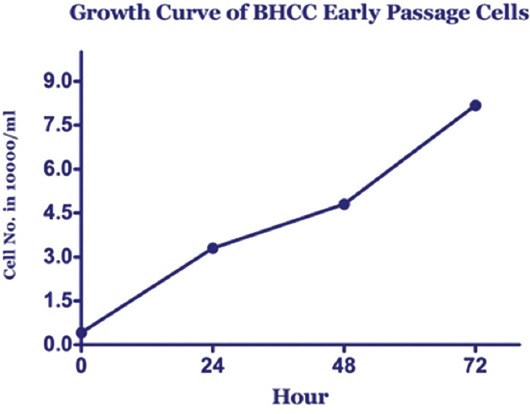
Growth curves of horn squamous cell carcinoma (bovine horn core carcinoma) early passage cells.

Population doubling time ascertained around 28.1 h (Figure-4), and cell viability ranged from 85% to 94%. The culture success rate was 90%.

### Transcriptomic comparison between SCC horn tissue and its early passage cells

The total number of genes differentially overexpressed in SCC horn tissue were 717 (8.40% of total genes expressed) compared to early passage cells; 150 genes (1.76% of total genes expressed) were differentially up-regulated which had more than 2-fold Log_2_ value with maximum value of 6.03-fold change. There were 746 genes (8.74% of total genes expressed) which had differential over-expression in early passage cells than SCC horn tissue, 248 genes (2.90% of total genes expressed) had more than 2-fold log_2_ value with maximum Log_2_ value 7.02. In this comparison, 5219 genes (~38% of total genes no., i.e., 14513 no.) showed no expression at the terms of FPKM in both the samples 1600 genes had more than 5 FPKM value in early passage cells.

### Genes overexpressed in SCC early passage cells and SCC horn tissue

Density plot and dispersion plot were derived for this comparison, respectively. Density plot assessed the distributions of FPKM scores across samples. Among the differentially expressed genes maximum genes had FPKM value between Log_10_ 1 and Log_10_ 2. Distribution of genes in SCC horn tissue ranged from Log_10_ 0.2 to Log_10_ 3.7 and for early passage SCC cells, it was Log_10_ 0.7 to Log_10_ 3.7. Dispersion plot showed normal dispersion of genes across samples. N-Myc downstream regulated 1, integrin alpha 6, TP53 apoptosis effector (PERP), eukaryotic translation initiation factor 4 A1 (A1EIF4A1), desmoplakin, etc., genes were up-regulated (up-to 2-fold FPKM value) in SCC horn tissue compared to SCC early passage cells. Up-regulated genes (up to 2-fold FPKM value) in horn SCC early passage cells compared to parental tissue were coiled-coil domain containing 69 (CCDC69), CCDC94, Sec61 gamma subunit (SEC61G), Paladin, *Hedgehog* (Hh) receptor patched homolog 1 (PTCH1), Armadillo repeat containing X-linked 2 and thioredoxin, etc.

GO category of the genes differentially expressed above 2 log_2_ fold change in SCC early passage cells compared to SCC horn tissue to be of calcium channel activity, calcium ion binding, protein phosphatase Type 2A activity and extracellular matrix (ECM) binding as per DAVID database (Table-1). The genes which were up-regulated in SCC horn tissue compared to its early passage cells showed major histocompatibility complex (MHC) Class I protein binding, MHC protein binding, procollagen proline 4-dioxygenase activity, peptidyl-proline dioxygenase activity, procollagen-proline dioxygenase activity, and protein disulfide isomerase activity.

**Table-1:** KEGG pathway of genes up in SCC early passage cells significantly over SCC horn tissue.

KEGG pathway	p value	Genes	Fold change	Fold enrichment	FDR
bta04350:TGF-beta signaling pathway	0.031289	MAPK1	+2.01994	4.113924	29.86879
		ROCK2	+2.16513		
		TGFBR1	+2.03972		
		PPP2CB	+2.30283		
		THBS1	+3.95799		
bta03010:Ribosome	0.037987	RPL32	+2.62569	3.869048	35.09439
		RPL23	+3.21069		
		RPS17	+3.94767		
		RPL3	+3.20979		
		RPL24	+2.62553		
bta05416:Viral myocarditis	0.06468	SGCG	+3.62476	4.262295	52.58867
		CASP9	+2.03964		
		MYH11	+3.03959		
		ITGB2	+3.03967		
bta05212:Pancreatic cancer	0.080806	VEGFC	+2.20969	3.880597	60.95311
		MAPK1	+2.01994		
		CASP9	+2.03964		
		TGFBR1	+2.03972		
bta04114:Oocyte meiosis	0.082876	CCNE2	+2.81728	2.981651	61.92363
		MAPK1	+2.01994		
		PPP2CB	+2.30283		
		PPP2R5E	+2.13926		
		ITPR2	+2.03959		
bta05200:Pathways in cancer	0.086841	CCNE2	+2.81728	1.930693	63.72096
		VEGFC	+2.20		
		MAPK1	+2.01994		
		PIAS4	+5.08415		
		CASP9	+2.03964		
		TGFBR1	+2.03972		
		MET	+2.03961		
		FGF10	+3.62461		
		PTCH1	+6.20952		
bta05010:Alzheimer’s disease	0.089018	MAPK1	+2.01994	2.484076	64.67474
		NDUFS5	+5.6258		
		NDUFB6	+3.62546		
		CASP9	+2.03964		
		COX5A	+2.04022		
		ITPR2	+2.03959		
bta04360:Axon guidance	0.094055	MAPK1	+2.01994	2.850877	66.79443
		ROCK2	+2.16513		
		MET	+2.03961		
		NTN4	+5.94653		
		SEMA3C	+2.62465		

KEGG=Kyoto Encyclopedia of Genes and Genomes, SCC=Squamous cell carcinoma, TGF=Transforming growth factor, FDR=False discovery rate, CASP9=Caspase 9, PPP2R5E=Protein phosphatase 2 regulatory subunit B epsilon

The percentage of genes which showed up-regulation in SCC horn tissue than SCC early passage cells was 1.76%. Genes up-regulated (≥2-fold) in SCC horn tissue as compared to horn SCC early passage cells were involved in biogenesis, apoptotic response and response to stimulus in biological processes; structural molecular activity and translation regulator activity in molecular function; cell part, organelle and macromolecular complex in cellular component and the up-regulated genes (≥2-fold) in horn SCC early passage cells were involved in cellular process, metabolic process, biological regulation in biological processes; catalytic activity, enzyme regulator activity, binding in molecular function; membrane, extracellular region in cellular component as per PANTHER database.

There was no pathway in 5 FDR limit, but the lowest FDR value was found at transforming growth factor (TGF) beta signaling pathway and ribosomal pathway for differentially up-regulated genes in SCC early passage cells compared to SCC horn tissue in Kyoto Encyclopedia of Genes and Genomes (KEGG) pathways (Table-2). Surprisingly, most of the genes which showed top fold change (within first 20) were not detected by DAVID during pathway analysis. Focal adhesion, ECM-receptor interaction, thyroid cancer, and pathways in cancer were shown by the genes which were up-regulated in SCC horn tissue than SCC early passage cells (Table-3).

**Table-2:** KEGG pathway analysis of significantly up regulated genes in SCC horn tissue in comparison to SCC early passage cells.

KEGG pathway	p value	Genes	Fold change	Fold enrichment	FDR
bta04510:Focal adhesion	0.003554	CDC42	−3.18266	3.489795918	3.85308
		ITGA6	−5.25806		
		ILK	−2.2332		
		COL6A2	−3.64219		
		PDGFRA	−2.10337		
		COL1A1	−2.81067		
		PPP1CB	−4.46812		
		THBS2	−2.81836		
		CTNNB1	−2.95534		
bta04512:ECM-receptor interaction	0.020151	CD44	−2.84292	4.704761905	20.12056515
		ITGA6	−5.25806		
		COL6A2	−3.64219		
		COL1A1	−2.81067		
		THBS2	−2.81836		
bta05216:Thyroid cancer	0.050381	NCOA4	−2.01925	8.142857143	43.47494112
		MYC	−3.83476		
		CTNNB1	−2.95534		
bta05200:Pathways in cancer	0.058738	HSP90AB1	−2.36404	2.096181047	48.72805735
		CDC42	−3.18266		
		HSP90AA1	−2.39775		
		ITGA6	−5.25806		
		NCOA4	−2.01925		
		PDGFRA	−2.10337		
		MYC	−3.83476		
		STAT3	−3.82663		
		CTNNB1	2.95534		
bta05412:ARVC	0.06167	ITGA6	−5.25806	4.342857143	50.4635378
		DSP	−4.84482		
		GJA1	−3.61259		
		CTNNB1	−2.95534		
bta04612:Antigen processing and presentation	0.066334	HSP90AB1	−2.36404	4.213219616	53.11403035
		HSP90AA1	−2.39775		
		PDIA3	−2.03351		
		HSPA8	−2.40875		
bta03040:Spliceosome	0.089962	PRPF8	−4.3062	2.892271663	64.66578307
		SNRNP200	−3.32965		
		DDX5	−2.27017		
		HSPA8	−2.40875		
		HNRNPU	−3.78478		

KEGG=Kyoto encyclopedia of genes and genomes, SCC=Squamous cell carcinoma, ARVC=Arrhythmogenic right ventricular cardiomyopathy, FDR=False discovery rate, ITGA6=Integrin alpha 6, ECM=Extracellular matrix

**Table-3:** GO of genes up regulated (≥2-fold) in SCC early passage cells compared to SCC horn tissue via DAVID.

Term	Count	FDR	%	p value
GO:0046872~metal ion binding	47	5.388398	21.17117	0.004134
GO:0043169~cation binding	47	6.774957	21.17117	0.005233
GO:0043167~ion binding	47	8.187385	21.17117	0.006369
GO:0019992~diacylglycerol binding	3	25.30314	1.351351	0.021584
GO:0050840~ECM binding	3	25.30314	1.351351	0.021584
GO:0000287~magnesium ion binding	7	34.49892	3.153153	0.031151
GO:0019838~growth factor binding	4	34.68459	1.801802	0.031356
GO:0015405~P-P-bond-hydrolysis driven transmembrane transporter activity	5	47.96599	2.252252	0.047687
GO:0015399~primary active transmembrane transporter activity	5	47.96599	2.252252	0.047687
GO:0008289~lipid binding	7	60.62826	3.153153	0.067344
GO:0005262~calcium channel activity	3	74.51316	1.351351	0.097192
GO:0005543~phospholipid binding	4	75.25716	1.801802	0.099191

Count denotes gene count. GO=Gene ontology, SCC=Squamous cell carcinoma, FDR=False discovery rate, ECM=Extracellular matrix

Genes up-regulated in SCC early passage cells compared to SCC horn tissue were involved in fibroblast growth factor signaling pathway, wnt signaling pathway, vascular endothelial growth factor signaling pathway, apoptosis signaling pathway and p53 signaling, epidermal growth factor receptor, cell cycle, inflammatory pathways mediated by chemokine and cytokine, etc., as per PANTHER database.

KEGG pathway of all genes, expressed in SCC early passage cells showed to be involved in focal adhesion, transforming growth factor TGF-beta signaling pathway, ubiquitin mediated proteolysis, pathways in cancer, prostate cancer mechanism within 5 FDR value (Table-4). KEGG pathways such as thyroid cancer, focal adhesion, small cell lung cancer, pathways in cancer, prostate cancer and spliceosome were shown to be involved when all the common genes (≥5 FPKM) between SCC horn tissue and SCC early passage cells compared in DAVID (Table-5). To unveil the genes involved in horn cancer pathogenesis, both *in-vivo* and *in-vitro* genes were mined from common pathways up to 5 FDR (Table-6).

**Table-4:** KEGG pathway of all genes expressing ≥5 FPKM in SCC early passage cells.

Term	Count	FDR	%	p value
bta04510:Focal adhesion	48	4.06E-06	3.292181	3.33E-09
bta04810:Regulation of actin cytoskeleton	40	0.042391	2.743484	3.47E-05
bta04350:TGF-beta signaling pathway	22	0.0641	1.508916	5.25E-05
bta04512:ECM-receptor interaction	21	0.091836	1.440329	7.52E-05
bta04520:Adherens junction	18	0.596801	1.234568	4.90E-04
bta04120:Ubiquitin mediated proteolysis	28	0.923001	1.920439	7.59E-04
bta05010:Alzheimer’s disease	30	2.493014	2.057613	0.002065
bta03010:Ribosome	19	3.336116	1.303155	0.002775
bta05200:Pathways in cancer	49	3.410855	3.360768	0.002838
bta05215:Prostate cancer	18	7.802359	1.234568	0.00663
bta05016:Huntington’s disease	30	7.998577	2.057613	0.006803
bta04670:Leukocyte transendothelial migration	22	8.071079	1.508916	0.006867
bta04114:Oocyte meiosis	21	12.05085	1.440329	0.01046
bta00640:Propanoate metabolism	9	15.5853	0.617284	0.013778
bta03050:Proteasome	11	17.36369	0.754458	0.015496
bta04270:Vascular smooth muscle contraction	20	17.64442	1.371742	0.015771
bta04530:Tight junction	22	19.73693	1.508916	0.017843
bta03040:Spliceosome	22	19.73693	1.508916	0.017843
bta05412:ARVC	14	20.06711	0.960219	0.018174
bta05211:Renal cell carcinoma	14	20.06711	0.960219	0.018174
bta04540:Gap junction	16	20.36185	1.097394	0.018471
bta05212:Pancreatic cancer	14	24.78525	0.960219	0.023053
bta05012:Parkinson’s disease	22	28.40347	1.508916	0.02699
bta05210:Colorectal cancer	16	31.81445	1.097394	0.030871
bta05414:Dilated cardiomyopathy	15	32.4247	1.028807	0.031584
bta04360:Axon guidance	20	32.69897	1.371742	0.031907
bta04110:Cell cycle	21	35.81972	1.440329	0.035663
bta05410:HCM	14	38.80693	0.960219	0.03942
bta04150:mTOR signaling pathway	11	39.72862	0.754458	0.040613
bta05222:Small cell lung cancer	15	40.92982	1.028807	0.042193
bta04720:Long-term potentiation	12	43.26753	0.823045	0.045355
bta04142:Lysosome	19	48.66196	1.303155	0.053134
bta05213:Endometrial cancer	10	55.76984	0.685871	0.064618
bta04666:Fc gamma R-mediated phagocytosis	15	59.04418	1.028807	0.070491
bta00520:Amino sugar and nucleotide sugar metabolism	9	60.46497	0.617284	0.073175
bta05220:Chronic myeloid leukemia	13	69.07887	0.891632	0.091639
bta00190:Oxidative phosphorylation	20	70.92517	1.371742	0.096207

Count denotes gene count. ARVC=Arrhythmogenic right ventricular cardiomyopathy, KEGG=Kyoto encyclopedia of genes and genomes, SCC=Squamous cell carcinoma, HCM=Hypertrophic cardiomyopathy, FPKM=Fragments per kilobase of exon per million, FDR=False discovery rate, ECM=Extracellular matrix

**Table-5:** KEGG pathway of all common genes (≥5 FPKM) in between SCC horn tissue and SCC early passage cells.

Term	Count	FDR	%	p value
bta04510:Focal adhesion	32	2.52E-06	4.878049	2.12E-09
bta04810:Regulation of actin cytoskeleton	26	0.014261	3.963415	1.20E-05
bta04512:ECM-receptor interaction	15	0.027243	2.286585	2.30E-05
bta05200:Pathways in cancer	31	0.450733	4.72561	3.81E-04
bta05215:Prostate cancer	13	1.366688	1.981707	0.00115989
bta05412:ARVC	11	1.968263	1.676829	0.00167511
bta04670:Leukocyte transendothelial migration	15	2.062266	2.286585	0.001755881
bta04520:Adherens junction	11	2.482973	1.676829	0.002118237
bta04120:Ubiquitin mediated proteolysis	15	10.18146	2.286585	0.009015052
bta04530:Tight junction	14	11.1892	2.134146	0.009957604
bta03040:Spliceosome	14	11.1892	2.134146	0.009957604
bta04350:TGF-beta signaling pathway	10	21.36772	1.52439	0.020069312
bta05213:Endometrial cancer	7	35.30036	1.067073	0.036055226
bta05216:Thyroid cancer	5	40.50522	0.762195	0.04284919
bta05414:Dilated cardiomyopathy	9	41.73228	1.371951	0.044529994
bta00310:Lysine degradation	6	44.89775	0.914634	0.049020477
bta05211:Renal cell carcinoma	8	45.27855	1.219512	0.049576494
bta04540:Gap junction	9	45.96247	1.371951	0.050584067
bta04110:Cell cycle	12	47.42947	1.829268	0.052785299
bta05222:Small cell lung cancer	9	48.09441	1.371951	0.053801616
bta05010:Alzheimer’s disease	14	53.2995	2.134146	0.062196631
bta05210:Colorectal cancer	9	56.59508	1.371951	0.067966842
bta03010:Ribosome	9	56.59508	1.371951	0.067966842
bta05410:HCM	8	61.65301	1.219512	0.077654962
bta04720:Long-term potentiation	7	64.27834	1.067073	0.083155068
bta04722:Neurotrophin signaling pathway	11	64.64354	1.676829	0.0839493

Count denotes gene count. HCM=Hypertrophic cardiomyopathy, FDR=False discovery rate, KEGG=Kyoto encyclopedia of genes and genomes, FPKM=Fragments per kilobase of exon per million, SCC=Squamous cell carcinoma, TGF=Transforming growth factor, ARVC=Arrhythmogenic right ventricular cardiomyopathy

**Table-6:** Genes common in pathways up to 5 FDR between SCC horn tissue and SCC early passage cells.

KEGG pathway term	FDR	Genes
bta04510:Focal adhesion	2.5165*E06	TLN1, COL3A1, ITGB1, CTNNB1, MYL9, VCL, ACTG1, CDC42, ITGAV, ILK, COL6A2, COL6A1, THBS2, PIK3R2, FN1, ACTB, COL4A1, ACTN4, PPP1CB, FLNB, FLNA, LAMA4, PPP1CA, CCND1, ITGA6, ITGA5, JUN, COL1A2, PDGFRA, RAP1A, PDGFRB, COL1A1, CRK
bta04810:Regulation of actin cytoskeleton	0.0142	RDX, PIP5K1A, ITGB1, MYL9, VCL, ACTG1, CDC42, EZR, GSN, ITGAV, MSN, FGF2, FN1, APC, PIK3R2, ACTB, ACTN4, PPP1CB, ARPC1A, PPP1CA, ITGA6, ITGA5, CFL1, PDGFRA, PDGFRB, CRK, PIP4K2C
bta04512:ECM-receptor interaction	0.0272	COL4A1, COL3A1, ITGB1, SDC1, LAMA4, ITGA6, CD44, ITGA5, ITGAV, COL6A2, COL1A2, COL6A1, COL1A1, THBS2, FN1
bta05200:Pathways in cancer	0.4507	HSP90AB1, TFG, MMP2, ITGB1, CTNNB1, CDC42, ITGAV, MYC, FGF2, FN1, APC, PIK3R2, COL4A1, HSP90AA1, EPAS1, CREBBP, SMAD4, CTNNA1, STAT3, LAMA4, HSP90B1, CCND1, CDKN1A, HIF1A, ITGA6, NCOA4, JUN, PDGFRA, PDGFRB, JAK1, CRK
bta05215:Prostate cancer	1.3666	HSP90AB1, HSP90AA1, CREBBP, CTNNB1, CCND1, HSP90B1, CDKN1A, ATF4, PDGFRA, CREB3L2, CREB3L1, PDGFRB, PIK3R2
bta05412:ARVC	1.9682	ACTB, ACTG1, ACTN4, ITGA6, ITGA5, ITGAV, LMNA, DSP, GJA1, CTNNA1, ITGB1, CTNNB1
bta04670:Leukocyte transendothelial migration	2.0622	ACTB, ACTN4, GNAI2, GNAI1, CTNNA1, MMP2, ITGB1, VCL, MYL9, CTNNB1, ACTG1, CDC42, EZR, RAP1A, MSN, PIK3R2
bta04520:Adherens junction	2.4829	ACTB, ACTG1, CDC42, PVRL1, ACTN4, PTPRF, CREBBP, SMAD4, CTNNA1, SNAI2, VCL, CTNNB1

ARVC=Arrhythmogenic right ventricular cardiomyopathy, FDR=False discovery rate, KEGG=Kyoto Encyclopedia of Genes and Genomes, SCC=Squamous cell carcinoma, ECM=Extracellular matrix

Genes that were uniquely expressed in SCC early passage cells as compared to SCC horn tissue showed involvement in metabolic and cellular process in biological processes; binding, catalytic activity in molecular function; heterotrimeric G protein signaling G_i_ alpha pathway, Huntington disease, endothelin signaling pathway, angiogenesis, interleukin signaling pathway, etc., in pathway as per PANTHER database.

High proliferative and antiapoptotic potential are related to the up-regulation of growth hormone receptor and calmodulins [31]. The top 20 genes which were found to be up-regulated in SCC early passage cells in comparison to SCC horn tissue were investigated to have roles in other cancers as well as SCC in human and domestic animals (Table-7) [32-61] and vice versa (Table-8) [62-95].

**Table-7:** Functions of highly expressed genes in SCC early passage cells in comparison to SCC horn tissue.

Gene ID (ENSBTAG)	Gene title	Name	FPKM EP	FPKM HCT	Log_2_ fold change	Roles and implications in cancer of human and other
00000002834	CCDC69	Coiled-coil domain containing 69	318.123	2.3446	+7.084	Expressed in various cancer cell lines such as HeLa, U2OS and MDA-MB-231, exogenous expression of CCDC69 in HeLa cells destabilized microtubules and disrupted the formation of bipolar mitotic spindles [32]
00000012830	CCDC94	Coiled-coil domain containing 94	842.151	10.503	+6.325	Avoids DNA damaging apoptosis in zebra-fish [33]
00000014971	SEC61G	Sec61 gamma subunit	4614.43	62.285	+6.211	Proto-oncogene required for tumor cell survival in GBM, involved in the cytoprotective ER stress–adaptive response to the tumor microenvironment [34]
00000008583	KIAA1274	Paladin	207.836	2.808	+6.209	Vascular-restricted expression in human brain, astrocytoma, and glioblastomas. Paladin expression is reactivated during pathological tumor angiogenesis in the - adult [35]
00000048213	PTCH1	Hh receptor patched homolog 1, Uncharacterized protein	92.8954	1.2552	+6.209	Inversely correlated with the metastatic potential of colon cancer cell lines, high expression associated with low Hh signaling [36]
00000003183	NTN4	Netrin 4	123.963	2.010	+5.946	Anti angiogenic effect, over expression could decrease tumor growth [37]
00000010232	NDUFS5	NADH dehydrogenase (ubiquinone) Fe-S protein 5, 15 kDa (NADH-coenzyme Q reductase)	1206.46	24.433	+5.625	Highly expressed in endometrial cancer [38,85]
00000019417	ARMCX2	Armadillo repeat containing, X-linked 2	145.59	2.950	+5.624	Might have a role in tumor suppression, role in development and tissue integrity [39]
00000021158	SATB1	SATB homeobox 1	161.255	3.7353	+5.431	High levels of SATB1 expression facilitate CRC and are associated with poor prognosis, promotes breast cancer metastasis, EMT marker in prostrate cancer [40]
00000003130	CHRNA3	Cholinergic receptor, nicotinic, alpha 3 (neuronal)	1615.84	43.60	+5.211	Polymorphism associated with high chance for NSCLC [41,85]
00000017633	EIF1AX	Eukaryotic translation initiation factor 1A, X linked	509.347	13.759	+5.210	Mutation is having protective role in uveal melanoma, over expressed in metastatic prostate cancer [42,43]
00000002428	PPA2	Pyrophosphatase (inorganic) 2	255.495	6.903	+5.209	Significantly increased in LNMPCa tissues, supplies increased energy requirement in metastasis - cells [44,45]
00000000753	PIAS4	Protein inhibitor of activated STAT, 4	582.593	17.174	+5.084	Necessary for proficient DNA repair of DSBs, promotes BRCA1 SUMOylation and DNA - repair [46,47]
00000013081	PSPH	Phosphoserine phosphatase	516.471	17.942	+4.847	Up-regulated in CRC, increased expression in non-small-cell lung cancer corresponds to clinical response. Suppression inhibited proliferation, tumor formation of MDAMB-468 and MCF10 cells respectively [48,49]
00000002953	TXN	Thioredoxin	3783.39	136.25	+4.795	Promote cell growth, induces VEGF, PTEN, angiogenesis and inhibit apoptosis in tumor - cells [50,51]
00000015522	MRPS31	Mitochondrial ribosomal protein S31	310.988	12.604	+4.624	Up-regulated in human breast cancer, CRC and found in 77% of all types of cancer [52,53,85]
00000045742	C5H12orf75	Chromosome 12 open reading frame 75	148.477	6.018	+4.624	Highly expressed in granulosa cells and membrane associated granulosa cells before ovulation in cattle [54]
00000009405	TRPC4	Transient receptor potential cation channel, subfamily C, member 4	51.582	2.091	+4.624	Highly expressed in NSCLC, LNCaP cells activating store operated channel calcium influx - factor [55,56]
00000008636	PDE4B	Phosphodiesterase 4B, cAMP-specific	41.5034	1.6824	+4.624	Highly expressed in diffuse large BCL, expression of it avoids CAMP mediated apoptosis. Induces angiogenesis and cell proliferation in lung cancer cell line [57,58]
00000008294	KCNJ2	Potassium inwardly-rectifying channel, subfamily J, member 2	35.2224	1.4278	+4.624	Expressed in medulloblastoma with poor clinical outcome, avoids apoptosis and induces cell proliferation in oral cancer also. Increased expression in papillary thyroid cancer [59-61]

EP=SCC early passage cells, HCT=SCC horn tissue, CAMP=Cyclic adenosine monophosphate, SCC=Squamous cell carcinoma, CCDC69=Coiled-coil domain containing 69, ER=Endoplasmic reticulum, GBM=Glioblastoma multiforme, Hh=Hedgehog, NADH=Nicotinamide adenine dinucleotide, CRC=Colorectal cancer, EMT=Epithelial mesenchymal transition, NSCLC=Non–Small Cell Lung Cancer, LNM=Lymph node metastasis, VEGF=Vascular endothelial growth factor, BCL=B-cell lymphoma, FPKM=Fragments per kilobase of exon per million

**Table-8:** Functions of highly expressed genes in SCC horn tissue in comparison to SCC early passage cells.

Gene ID (ENSBTAG)	Gene title	Name	FPKM HCT	FPKM EP	Log_2_ fold change	Roles and implications in cancer of human and other
00000000711	NDRG1	N-Myc downstream regulated 1	2001.28	30.4749	−6.03715	Regulated by androgens, acts as metastasis suppressor and negatively correlated with it, found to be down regulated in various cancers, prostate cancer [62,63]
00000017266	ITGA6	Integrin, alpha 6	835.447	21.8316	−5.2580	Prostate tumors persistently express ITGA6, linked to increased tumor cell invasion, migration, and metastasis. Increased adhesion in AML - cells [64,65]
00000020097	PERP	PERP, TP53 apoptosis effector	1624.07	47.026	−5.11001	Tumor suppressor. Loss induces tumorigenesis, cell survival, and desmosome loss by enhancing inflammatory set of genes in - SCCs [66,67]
00000000132	EIF4A1	Eukaryotic translation initiation factor 4A1	1613.66	54.0897	−4.8988	Associated with highly metastasizing melanoma. Overexpression is an early marker for metastasizing hepatocellular carcinoma and NSCLC [68,69]
00000015106	DSP	Desmoplakin	1837.68	63.9491	−4.8448	Loss of desmoplakin, a cell adhesion molecule, has been implicated in breast cancer metastasis [70]
00000047330	FABP5	Fatty acid binding protein 5 (psoriasis associated)	1255.27	51.7861	−4.5992	Involved in cell survival and growth, enhances cell proliferation and anchorage-independent growth in prostate and breast cancer - cells [71,72]
00000012447	PPP1CB	Protein phosphatase 1, catalytic subunit, beta isozyme	764.459	34.5396	−4.4681	Enhances proliferation and colony formation in leukemia cell line, expressed in 55 cancer cell lines [73,74]
00000010365	SQRDL	Sulphide quinone reductase-like (yeast)	1206.17	57.2255	−4.3976	Under expressed in ductal breast carcinoma, but down regulation reduce cell growth and induce apoptosis in breast cancer cell line [75,76]
00000011969	HSPB1	Heat shock 27 kDa protein 1	2770.43	137.28	−4.3349	Involved in DNA repair, recombination, anti-apoptotic activity in HeLa cells, in most of human cancers, high levels indicate presence of metastatic tissues. Low levels are associated with resistance [77,78]
00000011488	PRPF8	PRP8 pre-mRNA processing factor 8 homolog (*S. cerevisiae*)	230.594	11.6561	−4.3062	Associated with spliceosome pathway, tumor suppressor in myeloid malignancies [79,80]
00000012927	ALDOA	Aldolase A, fructose-bisphosphate, mRNA	1162.91	60.5281	−4.2639	Promote lung cancer metastasis, invasion capability [81,82]
00000015107	SLC16A1	Solute carrier family 16, member 1 (monocarboxylic acid transporter 1)	465.287	28.411	−4.0336	Positively associated with cell survival, negatively with mir-124 in medulloblastoma [83]
00000021035	CTSK	Cathepsin K, mRNA	917.7	56.0406	−4.0334	Inconsistent expression in horn cancer tissue in bovine, involved in Hh signaling and pre-osteoclast to osteoclast differentiation in breast cancer [84,86]
00000010793	CCDC80	CCDC80, mRNA	393.218	24.1296	−4.0264	Tumor suppressor, down regulated in thyroid carcinomas [87]
00000013315	ATP5B	ATP synthase, H+ transporting, mitochondrial F1 complex, beta polypeptide, mRNA	853.925	53.4692	−3.9973	Overexpressed and associated with poor survival in breast cancer. High ATP5B mRNA expression in ovarian cancer was associated with worse OS [88]
00000003418	MSN	Moesin (MSN), mRNA	339.836	22.2957	−3.93	High levels associated with poor breast cancer survival, by increased metastasis, invasion and EMT - changes [89]
00000008409	MYC	V-myc myelocytomatosis viral oncogene homolog (avian)	596.739	41.8222	−3.8347	Correlated with distant metastasis, aggressive breast cancer. Induces genome instability [90]
00000021523	STAT3	Signal transducer and activator of transcription 3 (acute-phase response factor), mRNA	566.044	39.895	−3.8266	Associated with increased angiogenesis, metastasis, immune signaling and inflammation in basal like breast - cancers [91,92]
00000008611	IGFBP4	Insulin-like growth factor binding protein 4	627.819	44.5065	−3.8182	Antagonist of wnt beta catenin signaling pathway, higher in metastatic RCC. Increases invasion, cell proliferation in glioma [93,94]
00000007606	HNRNPU	Heterogeneous nuclear ribonucleoprotein U (scaffold attachment factor A), mRNA	386.733	28.0595	−3.7847	Involved in spliceosome pathway in causing prostate cancer [95]

EP=SCC early passage cells, HCT=SCC horn tissue, NSCLC=Non-small cell lung cancer, ITGA6=Integrin, alpha 6, AML=Acute myeloid leukemia, ATP=Adenosine triphosphate, EMT=Epithelial-mesenchymal transition, RCC=Renal cell carcinoma, SCC=Squamous cell carcinoma, FPKM=Fragments per kilobase of exon per million, *S. cerevisiae*=*Saccharomyces cerevisiae*

## Discussion

In this study, we compared gene expression profiles of the two conditions, i.e., *in vivo* cancer tissue and *in vitro* cancer cells at their early passages. The growth and survival rate of SCC early passage cells were good and it grew for the first few passages without difficulties. The cellular compositions were homogeneous and were of morphological characteristics typical of squamous cell epithelium. These findings are more or less similar to previously described studies [31] that indicated that early passage cell cultures expressed genes similar to *in vivo* gene expression pattern. Hence, it could be used for *in vitro* investigation of transcriptomic alteration in cancers. Maximum value of differential gene expression in SCC early passage cells was 6.02-fold changes as compared to parental tissue. CCDC94 a dose-dependent modifier of the anti-apoptotic function of B-cell lymphoma 2 gene found to be up-regulated [96] in SCC early passage cells; PTCH1 overexpression might indicate invasive behavior of metastatic cells [97]; low Hh signaling [98] (Table-9). PTCH-1 overexpression in many epithelial-derived cancers correlates to overexpression of other “Hh pathway” members [99] and promotion of an alternate epidermal cell fate decision that potentiates SCC formation [100]. Netrin 4 overexpression might have control on reduced angiogenesis and metastasis [101]; high SATB homeobox 1 expression might have helped to promote cell cycle progression, proliferation, migration and increased invasive capability with strong expression of Vimentin (2750.61 FPKM) but low or lost E-cadherin (CDH1) expression - A pivotal event for epithelial to mesenchymal transition EMT [102]. EIF41A, X-linked gene overexpression along with EIF2A gene (fold change −0.56) downregulation shows improved cell proliferation as EIF2A gene is a negative regulator of protein translation, RPS7 gene overexpression (fold change −0.88) might have role in cancer cell cycle proliferation and cell cycle progression in BHCC early passage cells [103]. 14-3-3 gamma was not expressed in BHCC early passage cells denoting that 14-3-3 gamma might not be working at transcriptional level, but 14-3-3 theta which was found to be increased (fold change 0.30) might had a positive effect on tumor cell adhesion and growth [104]. In correlation to that Stratifin or 14-3-3 sigma was not expressed in BHCC early passage cells. Cyclin D1 (FPKM in BHCC early passage cells is ~86) which usually acts as an active switch for regulation of continuous cell cycle progression, had almost same expression in two samples, revealing the possible cycle chain in between these key players. Phosphoserine phosphatase [105]; inorganic pyrophosphates have a role in energy transduction, DNA replication and other metabolic processes that usually deregulate in cancer cells. It has been postulated that protein phosphatases are involved in the suppression of cellular growth and cancer development by antagonizing protein kinases in human cancers. Protein phosphatase 2 subunit B isoform alpha (PPP2R2A) is one of the four major Ser/Thr phosphatases and is a potential tumor suppressor gene [106], PP2, regulatory subunit B, epsilon isoform (PPP2R5E) expression are usually downregulated in cancer tissue and represses cell viability and growth promoting apoptosis in cells as a target of MicroRNA-23a (miR-23a) [107]. MiR-23a overexpression decreases PPP2R5E expression but as the cells were good and healthy by their phenotypes so we cannot support this hypothesis for our cell line. Glutaminase which indicates faster growth rate and change in Warburg effect [108] was increased (0.33-fold change) (not shown in table) in cells though, MYC oncogenic transcription factor expression in BHCC early passage cells was lower than BHCC tissue, and there was no expression of MiR-23a/b which are usually suppressed by MYC [109]. Solute carrier family 7A5, phosphoglycerate dehydrogenase decreased in cells, ACACA expression remained almost same, but ACLY expression was 1.5-fold lower in cells (Table-10). SERBP1 expression was also lower in cells by 1.5-fold. Moderate secretory carrier membrane proteins 3 expressions suggested a universal role in membrane traffic at the plasma membrane [110,111].

**Table-9:** Expression of genes that are usually altered in cancer and involved in cancer pathways.

Official gene symbol	SCC horn tissue FPKM	SCC early passage cells FPKM	Log_2_ (fold change)	Official gene symbol	SCC horn tissue FPKM	SCC early passage cells FPKM	Log_2_ (fold change)	Official gene symbol	SCC horn tissue FPKM	SCC early passage cells FPKM	Log_2_ (fold change)
Genes involved in TGF beta pathway [114]				Tumor suppressor genes [114]				Apoptosis [114]			
TGFB2	37.681	116.199	1.62466	PTCH1	1.25528	92.8954	6.20952	CDK2AP1	109.22	354.565	1.6988
TGFBR1	43.997	180.905	2.03972	ZFHX4	9.4298	15.5071	0.717634	CDK14	21.499	106.068	2.30264
TGFBI	381.88	71.3708	−2.41975	SDHB	298.285	108.237	−1.4625	CDKN1A	164.35	116.955	−0.4908
TGFB1I1	81.946	53.198	−0.62321	TP53INP1	109.129	15.4711	−2.81839	TNFRSF1B	17.236	85.0433	2.30275
CTGF	1237.8	1486.66	0.26426	TP53BP1	21.8765	28.4023	0.376625	TNFRSF1B	17.236	85.0433	2.30275
TGFB2	37.681	116.199	1.62466	WTIP	6.28321	38.751	2.62466	TNFRSF19	0	64.8309	∞
TERT	0	53.0329	∞	STK25	28.8784	59.3717	1.03979	WDR44	0	90.9718	∞
CDKs [114]				GSTK1	36.2615	111.844	1.62498	WDR45L	89.843	246.268	1.45474
CDKN1A	164.35	116.955	−0.4908	CTSC	337.901	48.4657	−2.80156	WDR48	50.640	44.6152	−0.1827
CDK16	59.044	58.2622	−0.01924	RB1	7.81432	16.0636	1.0396	APAF1	10.494	21.5734	1.03962
CDK2AP1	109.221	354.565	1.6988	RNF130	72.5241	137.638	0.92435	TNFAIP8L	36.440	89.9108	1.30295
CDK14	21.4991	106.068	2.30264	ZNF189	9.8045	30.2333	1.62462	TNFRSF1B	17.236	85.0433	2.30275
Genes highly expressed in cell, tumor [114]				RNF11	78.2997	307.308	1.9726	TNFRSF19	0	64.8309	Infinity
SPARC	5788.5	1127.85	−2.35963	RNF13	23.1794	122.535	2.40228	C1QTNF3	132.88	100.355	−0.40511
Genes expressed in immortal cell lines [114]				CDKN1A	164.35	116.955	−0.49082	TNFAIP8L1	27.853	34.3557	0.30271
TOP1	160.34	47.6561	−1.75045	SMAD4	105.153	102.396	−0.03833	APC pathway [114]			
PCNA	251.05	46.9208	−2.4197	Stability genes [114]				LRP12	84.539	38.6188	−1.13031
CDC26	0	276.369	Infinity	ATM	19.4638	9.2331	−1.07591	LRP4	7.1667	14.7322	1.03959
CDC2L1	52.649	30.9242	−0.76769	ATMIN	50.0996	19.9333	−1.32962	APC	14.067	8.26207	−0.7677
CDC27	37.956	16.7198	−1.18279	BRCA1	20.3512	15.6881	−0.37544	MYC	596.73	41.8222	−3.8347
Tumor suppressor genes [114]				Oncogenes [114]				CCND1	89.928	85.3383	−0.0755
APC	14.067	8.26207	−0.76779	MET	4.43146	18.2192	2.03961				
Tumor suppressor genes [114]				List of genes that are usually altered in cancer [115]				List of genes that are usually altered in cancer [115]			
EXT1	152.414	63.7274	−1.25801	KLF10	175.839	65.7242	−1.41976	AOX1	26.1803	37.9893	0.53711
EXT2	47.495	92.4998	0.96166	KLF5	207.845	134.928	−0.62332	BUB1	15.4024	23.7471	0.624594
GLi pathway [114]				KLF6	217.105	306.05	0.495374	NME1	264.994	163.488	−0.69677
EXT1	152.414	63.7274	−1.25801	TPX2	113.49	31.1076	−1.86723	PCDH18	6.99252	103.495	3.8876
EXT2	47.495	92.4998	0.96166	ACAT1	141.472	62.3266	−1.18259	PCDH17	3.49188	21.5346	2.62458
PTCH1	1.2552	92.8954	6.20952	CDC27	37.9565	16.7198	−1.18279	PCDH7	18.0213	17.098	−0.07587
CCND1	89.928	85.3383	−0.0755	CDC2L1	52.6498	30.9242	−0.76769	ABCA3	6.16494	30.4151	2.30263
PI3K pathway [114]				CDC26	0	276.369	∞	NMT1	58.3807	45.0079	−0.37531
SCAMP3	135.784	139.585	0.03983	MCM3AP	51.0153	28.6007	−0.83488	PRC1	97.9744	30.2115	−1.69731
NAMPT	19.696	69.4103	1.81721	SERBP1	565.676	213.062	−1.4087	PTTG1IP	207.906	122.119	−0.76765
AKTIP	61.4721	42.9791	−0.5163	NRBP1	140.308	129.802	−0.11229	SHMT1	20.3035	35.7767	0.817291
CTSC	337.90	48.4657	−2.80156	CIRBP	58.5565	57.7807	−0.01924	RRM2	81.5659	61.1926	−0.41461
LAMTOR5	65.978	203.559	1.62537	CDH13	11.6941	48.0794	2.03963	TOP1	160.345	47.6561	−1.75045
LAMTOR4	0	207.588	∞	COL4A1	156.377	60.2731	−1.37544	SCFD1	24.4279	129.136	2.40229
AEBP1	269.61	95.0153	−1.50469	ENO1	1156.36	581.125	−0.99266	NAP1L4	142.404	83.6444	−0.76765
RPS6KA4	26.142	29.3143	0.165186	RBFOX2	68.9153	28.3343	−1.28228	SPP1	909.522	965.956	0.086848
RPS6KB1	69.345	183.303	1.40236	FOXN3	47.44	22.9456	−1.04788	CCNE2	19.4415	137.03	2.81728
RPS6KC1	27.447	19.9144	−0.46289	FOXJ2	29.0679	29.878	0.039658	CCNY	154.802	65.8485	−1.2332
BCL2L13	12.845	118.832	3.20963	PRKAR1A	534.133	67.9277	−2.97513	TRMT10A	4.72355	58.2622	3.62462
Oncogenes [114]				PRKAR2A	141.262	62.2342	−1.18259	ARHGAP24	13.8698	68.4312	2.30271
METTL13	8.588	35.31	2.03968	TGFBI	381.889	71.3708	−2.41975	New cancer genes [115]			
PDGFRA	55.2793	12.8642	−2.10337	TGFBR1	43.9979	180.905	2.03972	ITM2B	731.712	364.724	−1.00447
Hh pathway [114]				THBS2	295.379	41.8762	−2.81836	NUP205	37.2026	55.6184	0.580157
ARNTL	5.52379	39.7419	2.84693	CKAP2	70.1512	112.865	0.686055	FAT1	168.926	148.005	−0.19075
				UBE2C	150.04	142.406	−0.07533	ITM2C	95.1238	45.129	−1.07575

SCC=Squamous cell carcinoma, FPKM=Fragments per kilobase of exon per million, TGF=Transforming growth factor

**Table-10:** Genes commonly deregulated in cancer.

Official gene symbol	SCC horn tissue FPKM	SCC early passage cells FPKM	Log_2_ (fold change)	Official gene symbol	SCC horn tissue FPKM	SCC early passage cells FPKM	Log_2_ (fold change)
Genes up regulated in most cancers [110]				IQGAP3	13.3333	14.9501	0.16512
				ZBTB11	51.1437	93.4543	0.86970
IPO7	330.60	70.3061	−2.2333	RPN2	308.245	578.589	0.90846
FKBP10	125.032	34.2715	−1.86721	IPO4	24.7048	50.7859	1.03964
PRC1	97.974	30.2115	−1.69731	FARP1	25.0428	57.9149	1.20954
FNDC3B	79.1106	25.3438	−1.64224	TMEM41B	35.6942	82.5496	1.20957
ILF3	79.5314	25.8148	−1.62332	TTLL4	18.5496	45.7588	1.30266
ACLY	121.74	41.7097	−1.54534	GEMIN6	66.7981	164.81	1.30292
ADAM12	69.750	29.6667	−1.23336	CALU	213.254	563.68	1.4023
PSMB2	315.665	139.101	−1.1822	SNX10	17.7203	72.8583	2.03969
EIF2AK1	56.6175	31.7431	−0.8348	RBAK	12.6522	93.6353	2.88766
NME1	264.99	163.488	−0.6967	EPRS	0	36.3044	∞
ADAM10	58.022	39.761	−0.5452	PGK1	0	277.607	∞
ANP32E	196.546	138.547	−0.5044	WISP2	0	257.533	∞
HNRPLL	43.4377	31.5166	−0.4628	Commonly down regulated genes in most cancers [110]			
FAM49B	148.32	107.624	−0.4627	ERBB2IP	54.7664	56.29	0.03958
EIF2S2	396.41	344.378	−0.2030	DHRS4	64.1568	65.9521	0.03981
KDELR3	213.373	202.472	−0.0756				
SPP1	909.522	965.956	0.08684				
UTP18	44.7713	50.2058	0.16527				
ZBTB1	42.3114	49.7028	0.23228				

SCC=Squamous cell carcinoma, FPKM=Fragments per kilobase of exon per million

Cytoplasmic serine hydroxymethyltransferase 1 (SHMT1) and thymidylate synthase genes of the *de novo* thymidylate biosynthesis pathway were found to be increased in early passage cells than BHCC tissue, but SHMT2 was not expressed in cells [110,112,113]. Tumor protein 53-induced nuclear protein 1, apoptosis activating factor-1 was found to be increased in BHCC early passage cells (>1-fold change) along with effector genes such as caspase 6 (CASP6) and caspase 9 (CASP9) (>2-fold change) but in contrast cytochrome C was not found to be expressed and the genes CASP3, CASP8 were not detected [114]. The above discussion denotes a number of key players in pathogenesis of SCC of horns in bovines which showed resemblance with human cancer studies in expression profiling.

## Conclusion

The signaling pathway investigation in this first culture based approach revealed that many of the cancer-related pathways reported in the literatures for other carcinomas may also be held responsible for SCC of horn in bovines. Cells from bovine horn SCC surgical specimens may be adapted *in vitro* with high efficiency, independently from any clinicopathological characteristics.

Low-passage horn cancer cell lines would still closely reflect the phenotype of the horn cancer cells *in vitro* bypassing the obstacle for obtaining more detailed insights into the diversity of phenotypic and molecular changes occurring in horn cancer cells. Our result based on the pathway analysis suggested that primary culture of horn cancer *in-vitro* may serve as the model for SCC of horns in cattle.

This transcriptome-based approach demonstrates that epithelial cultures isolated from primary horn SCC retain complex characteristics of the malignant tissue. Thus, the opportunity for basic and clinical application of functional cells derived from SCC horn tissue, instead of a few immortal cell lines should not be missed.

## Authors’ Contributions

SS: Carried out laboratory experiment and written manuscript as part of MVSc. in Animal Genetics and Breeding. RSJ: Helped in manuscript correction. CGJ: Conceptualized the project. AKP: Helped in tissue culture work. RKS: Helped in tissue culture work. NP: Helped in bioinformatics work. SJJ: Helped in NGS work. SK: Helped in manuscript writing. BR: Helped in bioinformatics work. PGK: Helped in NGS work and sample collection. DNR: Helped in manuscript correction and improvement. All authors read and approved the final manuscript.
